# Simulation suggests that rapid activation of social distancing can arrest epidemic development due to a novel strain of influenza

**DOI:** 10.1186/1471-2458-9-117

**Published:** 2009-04-29

**Authors:** Joel K Kelso, George J Milne, Heath Kelly

**Affiliations:** 1 School of Computer Science and Software Engineering, University of Western Australia, Perth, WA, Australia; 2 Epidemiology Unit, Victorian Infectious Diseases Reference Laboratory, Victoria, Australia; 3 School of Population Health, University of Melbourne, Victoria, Australia

## Abstract

**Background:**

Social distancing interventions such as school closure and prohibition of public gatherings are present in pandemic influenza preparedness plans. Predicting the effectiveness of intervention strategies in a pandemic is difficult. In the absence of other evidence, computer simulation can be used to help policy makers plan for a potential future influenza pandemic. We conducted simulations of a small community to determine the magnitude and timing of activation that would be necessary for social distancing interventions to arrest a future pandemic.

**Methods:**

We used a detailed, individual-based model of a real community with a population of approximately 30,000. We simulated the effect of four social distancing interventions: school closure, increased isolation of symptomatic individuals in their household, workplace nonattendance, and reduction of contact in the wider community. We simulated each of the intervention measures in isolation and in several combinations; and examined the effect of delays in the activation of interventions on the final and daily attack rates.

**Results:**

For an epidemic with an R_0 _value of 1.5, a combination of all four social distancing measures could reduce the final attack rate from 33% to below 10% if introduced within 6 weeks from the introduction of the first case. In contrast, for an R_0 _of 2.5 these measures must be introduced within 2 weeks of the first case to achieve a similar reduction; delays of 2, 3 and 4 weeks resulted in final attack rates of 7%, 21% and 45% respectively. For an R_0 _of 3.5 the combination of all four measures could reduce the final attack rate from 73% to 16%, but only if introduced without delay; delays of 1, 2 or 3 weeks resulted in final attack rates of 19%, 35% or 63% respectively. For the higher R_0 _values no single measure has a significant impact on attack rates.

**Conclusion:**

Our results suggest a critical role of social distancing in the potential control of a future pandemic and indicate that such interventions are capable of arresting influenza epidemic development, but only if they are used in combination, activated without delay and maintained for a relatively long period.

## Background

Concern exists that the avian H5N1 influenza virus may become readily transmissible between humans, leading to a pandemic with significant mortality [1].

Social distancing interventions, such as school closure, reducing workplace numbers, reducing social and community contacts, and increasing home isolation are embedded within the pandemic influenza preparedness plans of most countries [2-4] and appear in current WHO recommendations. Social distancing interventions are important as they represent the only type of intervention measure guaranteed to be available against a novel strain of influenza in the early phases of a pandemic. The goal of these interventions is to reduce the overall illness attack rates and the consequential excess mortality attributed to the pandemic, and to delay and reduce the peak attack rate, reducing pressure on health services and allowing time to distribute and administer antiviral drugs and, possibly, suitable vaccines.

Modelling [5] has suggested that early interventions which increase social distancing may postpone the time to reach peak attack rates and limit the total number of cases and deaths attributed to pandemic influenza. This theoretical work has recently been supported by archival studies of excess deaths attributed to the 1918–19 pandemic in the largest US cities [6] and by the work of [7] for an Australian city. While these studies show that the historically implemented measures were not effective in preventing any local epidemics, they do show a strong correlation between the delay in introduction of intervention measures and excess mortality (both total and peak).

However, the potential impact and possible limitations of social distancing measures are not fully understood. An evaluation of the evidence base for non-pharmaceutical interventions concluded that there is a general lack of scientific evidence or expert consensus for school closure, workplace closure or banning of public gatherings during a pandemic [8].

Epidemiological simulation models have been used to analyse the effects of alternative containment measures. Various studies have simulated influenza pandemics at the scale of the whole world [9], whole countries [10-13], and individual communities [14,15]. A picture that emerges from a comparison of such simulation studies is that the predicted efficacy of social distancing intervention measures can depend strongly on the particular assumptions made about the operation of each intervention [15]. For example, little can be predicted about the outcome of school closure without specifying the contact behaviour of students when schools are closed, the timing of introduction of closure, or the other intervention measures that are concurrently in effect.

The purpose of this study was to extend the scope of simulations of social distancing interventions in an influenza pandemic by examining several important assumptions which have been not previously studied in a systematic way. We present results from an examination of the *timing-of-activation *and *combination *of social distancing interventions to determine how these factors impact their effectiveness, and thus to inform policy decisions regarding reactive strategies for mitigating the effects of an influenza pandemic.

## Methods

In previous work, we constructed a detailed, individual-based model of a real community in the south west of Western Australia (Albany) with a population of approximately 30,000, and applied the model to conduct simulations of the spread of pandemic influenza – full details of the model can be found in [15].

We used census data and state and local government data to construct a population of virtual individuals and households that matched the age and household structure of the real town. Individuals were also grouped into a number of "contact hubs" such as schools, child care facilities, adult educational facilities, workplaces and the regional hospital. Additional random contact in the community was modelled, with contacts biased towards meetings between individuals with nearby household locations.

Each simulation proceeded in a sequence of 12-hour cycles. During each cycle, a nominal location of each individual was calculated; taking into account the type of cycle (day or night, weekend or weekday), the contact hub the individual was a member of (if any), and the infection status of the individual and so forth. Individuals occupying the same household or contact hub during the same cycle were deemed to come into potentially infectious contact.

When a susceptible and infectious individual came into contact, a probability of infection transmission was calculated, based on the underlying infectivity of the viral strain, the age of the susceptible individual, and the progress and severity of the infection of the infectious individual. Influenza infection was assumed to proceed for 6 days, with 1 day latent, 1 day asymptomatic and infectious, followed by 4 days infectious (either symptomatic or asymptomatic). Although there is little evidence for spread from asymptomatic subjects for pandemic influenza, we adopted the conservative assumption that the proportion of individuals experiencing asymptomatic infection matched that of seasonal influenza.

Full details of the methodology and data sources used to select the various model parameter values can be found in [15], and the resulting parameter value settings are recorded in Supporting Information Text S1 of that publication. Since the infectivity of an epidemic arising from a new strain of influenza is uncertain, we simulated epidemics with basic reproduction numbers (R_0_) of 1.5, 2.5 and 3.5, which, assuming no intervention, gave final symptomatic attack rates of 33%, 65% and 73% respectively. Characteristics of these baseline epidemics are given in Table 1.

**Table 1:** Characteristics of baseline epidemics

**Characteristic**	**R**_0_					
	1.5		2.5		3.5	
	
	Mean	S.D.	mean	S.D.	mean	S.D
Final infection rate (%)	39.7	1.47	79.7	0.52	91.2	0.24
Final attack rate (%)	33.3	1.2	64.8	0.41	73.2	0.29
Peak symptomatic population (%)	4.8	0.41	25.7	0.55	43.0	0.71
Peak daily attack rate (per 10,000)	87	8	481	21	856	32
Peak attack day	57	5.6	29	2.0	20.	1.7
Serial interval	2.97	0.01	2.74	0.01	2.45	0.01

This table gives characteristics of simulated baseline epidemics (that is, epidemics where no intervention measures were applied) with R_0 _values of 1.5, 2.5 and 3.5. The mean and standard deviation (S.D.) are for 40 independent randomly seeded simulation runes.

For the study described in this paper we extended that model to allow *delayed *introduction of social distancing intervention measures, and conducted a new simulation experiment series. We simulated four different social distancing intervention measures. For each measure (and for several combinations of simultaneously applied measures), we simulated the effect of introducing each intervention measure (and several combinations of simultaneously applied measures) at different points in time, ranging from 0 to 8 weeks after the introduction of the first infectious case into the community. Once introduced, it was assumed that interventions continued until the end of the simulation.

The four intervention measures simulated were as follows:

### School closure

We assumed that when schools were closed, students and teachers spent weekday daytime cycles at home rather than at school. This meant that no contact took place at that school, but that these individuals would contact any other individuals present in their household during the day. We also assumed that if school closure would result in a child being present in a household alone, one adult from the household stayed home (and did not make hub contacts).

### Increased case isolation

When the *increased *case isolation intervention was in effect, there was a 90% (100% for children) chance that, upon become symptomatic, an adult (or child) would withdraw to their household for the duration of their infection (in the no-intervention case these probabilities were 50% for adults and 90% for children). We assumed that withdrawn individuals made only household contacts thereafter.

### Workplace non-attendance

When this measure was in effect, each person attending a workplace had a 50% chance each day of staying home instead of attending the workplace. Individuals staying at home made contacted all other individuals at home during the day.

### Community contact reduction

When this measure was in effect, individuals made half as many community contacts with other individuals per day.

## Results

### Impact of intervention activation delay on epidemic attack rates

**Figure 1:**
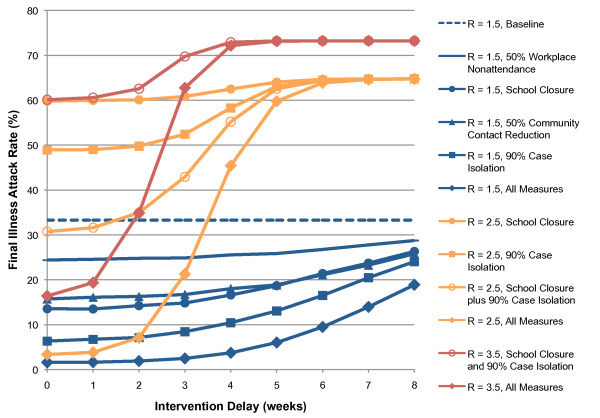
**Relationship between intervention activation delay and final illness attack rates**. Results for five different intervention strategies are shown for R_0 _= 1.5 (blue); four strategies are shown for R_0 _= 2.5 (orange); two strategies are shown for R_0 _= 3.5. All data points are averages of 40 randomly seeded simulation runs; standard deviation each 40-run set was < 1.4% of the population.

We use a final symptomatic attack rate of < 10% as a criterion for determining that an epidemic due to a novel pandemic strain of influenza has been prevented. For an epidemic with an R_0 _value of 1.5, we found that the only single intervention measure capable of preventing an epidemic was the 90% case isolation measure, and only if applied within 3 weeks. We found that a combination of all four social distancing measures could reduce the final attack rate from 33% to 9% if introduced within 6 weeks from the introduction of the index case. If applied pre-emptively, anticipating the arrival of the index case into the otherwise uninfected community, this combination of measures reduces the total attack rate to 1.6%, with a correspondingly significant reduction in attendant mortality rates. Figure 1 shows the relationship between final attack rates and intervention delays for each intervention measure on its own and the combination of all measures.

In contrast, for an epidemic with an R_0 _value of 2.5 the combination of all intervention measures must be introduced within 2 weeks of the index case to prevent an epidemic developing; delays of 2, 3 and 4 weeks resulted in final attack rates of 7%, 21% and 45% respectively (see Figure 1). No single intervention measure could reduce the final attack rate to less than 48%, even if activated pre-emptively. While not controlling the epidemic, the combination of school closure plus 90% case isolation approximately halved the final attack rate (from 65% to 31%), illustrating the value of layering multiple intervention measures, especially for high values of R_0_.

For an epidemic with an R_0 _of 3.5, perhaps considered to be a worst-case scenario, our results indicate that the combination of all interventions was unable to reduce the final illness attack rate to less than 10% and unable to prevent an epidemic occurring. However, the rapid activation of measures may significantly arrest epidemic development, giving final attack rates of 16%, 19% and 35% if activated pre-emptively or with a 1 or 2 week delay, respectively.

**Figure 2:**
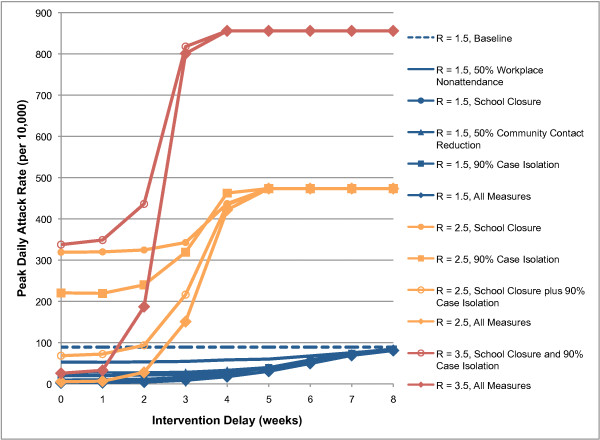
**Relationship between intervention activation delay and peak daily illness attack rates for various intervention strategies**. Results for R_0 _= 1.5 (blue), R_0 _= 2.5 (orange), and R_0 _= 3.5 (red) are shown.

We found a similar effect of intervention delay on peak daily attack rates (see Figure 2). For an epidemic with an R_0 _of 1.5, any of the intervention measures except 50% workplace non-attendance reduced the peak daily attack rate from 90 cases per 10,000 to below 35 if introduced within 4 weeks. For an R_0 _of 2.5, only the combination of all measures applied within 2 weeks could reduce the peak daily attack rate from 474 to below 35 cases per 10,000. Delays of 2, 3 or 4 weeks resulted in peak daily attack rates of 28, 151 or 422 cases per 10,000 respectively. To put this into context, if it is assumed that 1% of cases require hospitalisation [16], delays of 2, 3 or 4 weeks would require the 120-bed regional hospital in the 30,000 person community to handle 0.84, 4.5 or 12.7 influenza admissions per day at the peak of the epidemic, respectively. For an epidemic with an R_0 _of 3.5, the situation is even more stark: the combination of all measures applied within one week reduced the peak daily attack rate from 856 to 33; delays of 2 or 3 weeks resulted in peak daily attack rates of 187 or 801 cases per 10,000 respectively. Figure 2 shows the relationship between peak daily attack rates and intervention measure activation delay.

**Figure 3:**
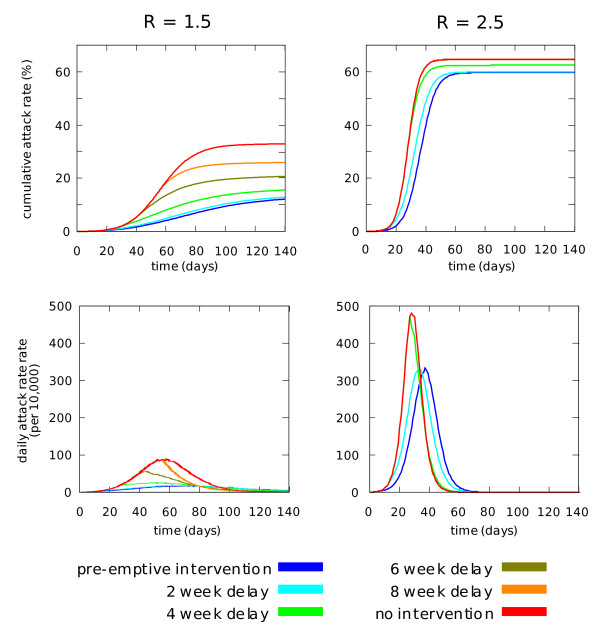
**Epidemic curves for school closure for a range of activation delays**. Cumulative (top) and peak daily (bottom) attack rates are shown for epidemics with unmitigated R_0 _values of 1.5 (left) and 2.5 (right).

In order to illustrate the effect of delays in activation of intervention measures on the time course of an epidemic, Figure 3 shows cumulative and daily attack rate epidemic curves for school closure, for delays of between 2 and 8 weeks. The figure clearly shows the contrast between epidemics with R_0 _values of 1.5 and 2.5. In the former case the rate of infection peaks on day 58; pre-emptive school closure is capable of making large reductions in final and peak daily attack rates, and each additional delay of 2 weeks steadily reduces the effectiveness of the intervention. In the latter case the epidemic peaks on day 28; school closure does not make a large reduction in final attack rate even if applied pre-emptively, and reduction in peak daily attack rate declines suddenly between delays of 2 and 4 weeks.

### Relationship between intervention delay and observed case trigger thresholds

We have examined the effect of delaying intervention in terms of the time between the first infected individual appearing in the population and the activation of intervention measures. In reality, it is unlikely that the first case of pandemic influenza in a community would be identified in a timely fashion. One observable measure of epidemic progress used in public heath planning is the proportion of the population known to have been infected, as estimated by the number of reported cases. Table 2 relates the number of observed cases to a corresponding activation delay for a range of intervention trigger thresholds, assuming that only 50% of symptomatic cases are reported.

**Table 2:** Diagnosed case thresholds and intervention trigger timings

Threshold	Cases	R_0_			
		1.5	2.0	2.5	3.5
		
		Delay until threshold reached (days)			
0.05%	15	**9**	**7**	**6**	5
0.1%	30	**13**	**9**	**8**	6
0.5%	150	**24**	**16**	**13**	10
1.0%	300	**30**	**19**	15	11
2.0%	600	**37**	23	18	13
4.0%	1200	46	27	21	15
8.0%	2400	58	32	24	17

This table shows a range of case count trigger thresholds and the consequent delay in intervention corresponding to each threshold. The boldface delays are those for which the final illness attack rate is < 10%, if all four intervention measures are activated at that time and continued indefinitely.

For a case to count towards the intervention threshold it is assumed that the following sequence of events occurs:

a) The individual becomes infected.

b) The individual experiences symptomatic infection.

c) They present to a health care professional in a monitoring scheme.

d) The infection is correctly diagnosed as pandemic influenza.

e) The case is reported to the monitoring scheme.

We assume a 50% ascertainment efficiency, which is the conditional probability that e) occurs, given that both a) and b) have occurred. We assume that there are no false-positive reports of pandemic influenza.

### Impact of interventions on age-specific attack rates

**Figure 4:**
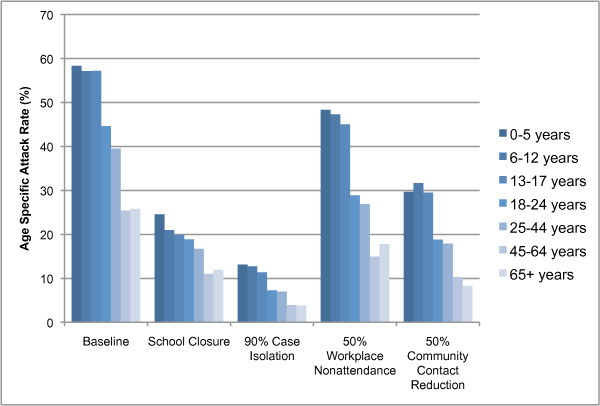
**Age-specific attack rates for social distancing interventions**. Final attack rates are shown for each of 7 age groups for a baseline (unmitigated) epidemic, and for epidemics mitigated by 4 intervention measures. An R_0 _value of 1.5 is assumed; interventions are assumed to be applied pre-emptively.

**Figure 5:**
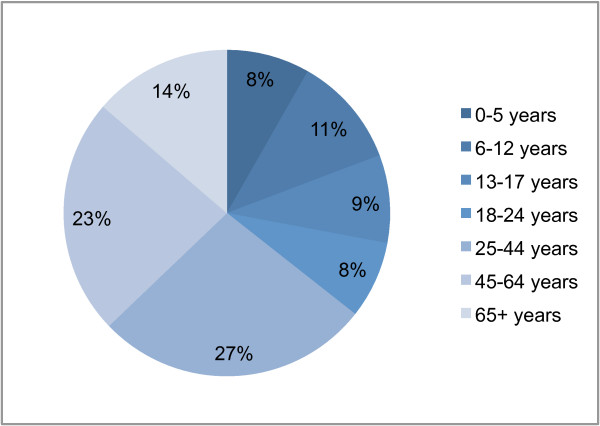
**Age structure of simulated population**. The percentage of the total simulated population (29350) of each of the 7 age groups is shown.

Our results showed that the simulated intervention measures affected age groups differentially. The interventions that caused the largest deviation from the baseline age-specific attack rate profile were school closure, which caused a larger proportional reduction in the attack rate of the school age range (6–17 years) compared to other age groups; and workplace nonattendance, which resulted in a proportionally larger reducing the attack rate of the 18–64 age group. The age-specific attack rates for the baseline epidemic (which was calibrated to resemble that of seasonal influenza), and epidemics mitigated by each of the four intervention measures are shown in Figure 4. It is interesting to note that the 50% workplace nonattendance measure and the school closure measure directly target approximately the same sized sub-population each day: half of the 18–64 age group, or 29%; compared to all of the 0–17 age group, or 28% (Figure 5 shows that age structure of the simulated population). Despite this fact, school closure has the greater effect, showing that in our model school children are disproportionally responsible for transmitting infection.

## Discussion

We consider social distancing interventions for a number of reasons; to better understand the effect which individual social distancing measures have on the attack rate and consequential mortality rate; to determine their sensitivity to the time of activation; to address scenarios where supplies of antiviral drugs and suitable vaccines are in limited supply, either due to an outbreak occurring in a country with little access to these resources drugs, due to logistical delays in their distribution and administration or due to the unavailability of an appropriately "typed" vaccine, particularly at the early states of a pandemic.

Antiviral drugs and social distancing interventions share several common characteristics: neither confers long-lasting immunity, and both deplete limited resources once in operation (a drug stockpile on one hand, and public patience on the other). The use of antiviral drugs is a core feature of the pandemic preparedness plans of many countries, such as the United States, the United Kingdom, and Australia [4,3,2]. Their use in an influenza pandemic is however an untested strategy: more experience is needed to determine their likely effectiveness and optimal use, especially given the possibility of the development of antiviral drug resistance during or prior to a pandemic [17-19]. It therefore seems prudent to consider social distancing interventions as an alternative or complement to antiviral-based strategies.

Social distancing interventions are important as they represent the only type of intervention measure guaranteed to be available against a novel strain of influenza in the early phases of a pandemic. They may be readily activated and thought of as a first line of defence in developing and developed countries alike. For the purposes of our study we have assumed that once intervention measures have been activated they continued indefinitely. The final attack rates reported thus represent ideal scenarios: preliminary results of additional research indicate that interventions would need to continue for approximately 5 months to prevent an epidemic with an R_0 _of 2.5, which is clearly unrealistic. While long-term imposition of socially and economically disruptive measures is not possible, social distancing interventions may be used to buy time for the establishment of an antiviral containment programme and/or the distribution of a vaccine [4,3,2].

The historical record indicates that social distancing measures may be implemented, relaxed and sometimes re-implemented [6,7,20]. Other models have investigated the optimal timing for rescinding and re-implementing social distancing interventions [21]. However our results show what may be expected of social distancing interventions when implemented early and maintained indefinitely (or in practical terms, until an effective vaccination programme has been completed); and we establish maximum activation delays allowable if such interventions are to fulfil their potential.

The results are applicable to industrialised populations and are possibly not applicable to developing countries having lower population mobility and/or higher population densities. In such countries we may find higher daily contact rates and hence reduced opportunities for limiting contact and in achieving isolation in the household by non-pharmaceutical means.

When comparing our results to those of other models, differences arise which may be due to alternative assumptions being made regarding the demographics and contact behaviour of the population, and to different assumptions regarding interventions and the methods for deploying them. However, we are able to comment at a general level on how our results relate to that of methodologically similar studies.

The work of Glass et al [5] most closely resembles that of ourselves, whereby they utilise a population of 10,000 individuals and, like us, examine only non-pharmaceutical interventions. Their model represents the estimated structure and contact patterns of a synthetic town in the USA; it is unclear to us how the differences in the detail modelled, between an actual population (Albany, Australia in our case) and this synthetic town affect the quality of the results obtained. The results coincide well for an R_0 _of 1.5 to 1.6, when considering school closure as the only intervention. With an R_0 _of 2.5 and all non-pharmaceutical interventions activated together, the results in [5] suggest a reduction in the illness attack rate in the range 10% to 20%, depending on variation of the contact patterns assumed. By contrast, the results presented here suggest that an attack rate of as low as 3% may result if all interventions are activated optimally, that is within the first week of the arrival of an outbreak-producing index case and held indefinitely. One difference which may explain this variance is that Glass et al assume a threshold of 10 diagnosed cases in a school before closure is effected compared with the optimal strategy adopted by ourselves. This again highlights the significance of rapid intervention if we are to prevent an epidemic, or to substantially reduce its rate of growth.

In comparing whole-country models such as that produced for the USA by Germann et al [13], the only control interventions which can be directly compared is that of school closure in isolation. The simulated attack rate for unmitigated epidemics with R_0 _= 1.5/1.6 are reduced to 1% and 13%, comparing the Germann et al result with that presented here. What is perhaps more interesting is that for R_0_'s in the range 1.9 to 2.4 significant reductions in attack rates to a level where an epidemic may be prevented are only achieved by Germann et al by combining non-pharmaceutical interventions with either targeted antiviral prophylaxis or vaccination, with the exception of child-first vaccination for an R_0 _of 1.9. Our results suggest that for R_0_'s up to and including 2.5 epidemics may be prevented by combined non-pharmaceutical interventions alone, provided they are activated immediately and are sustained indefinitely. Given the logistics of vaccination and antiviral drug deployment it is highly likely that non-pharmaceutical interventions may be activated more rapidly and our results suggest a similar ability to prevent epidemic development as that achieved by a combined pharmaceutical/non-pharmaceutical strategy.

The need to react rapidly when activating interventions is also highlighted by Ferguson et al [12] where antiviral treatment is very sensitive to initial delays of 24 hours, due to the use of an early, peaked infectiousness function. This contrasts with our more abstract flat infectivity profile. Single non-pharmaceutical interventions, such as school closure or home quarantine, are shown to have little impact for R_0 _in the range 1.7 – 2.0, whilst more significant reductions are suggested in the results that we derive. Similarly, school closure and 50% workplace reduction has less effect (an approximately 4 percentage point reduction in attack rate) in the Ferguson et al model compared to our results, where the suggested reduction in attack rate is of the order of 20 percentage points. While there are clear differences in assumptions between the two models and direct comparison is difficult, key factors may be their requirement to diagnose one case in a school before closure is effected, and their assumption that additional community contact occurs when schools are closed.

The range of modelling estimates for the potential effectiveness of social distancing interventions such as school closure is considerable, and may stem from the range of modelling assumptions about the operation of school closure and associated behavioural changes of individuals [15]. Observations of actual school closures do not seem to provide conclusive evidence on the effectiveness of school closure. Based on observations made during a teacher's strike in Israel, it was estimated by Heymann et al [22] that diagnoses of respiratory infections decreased by 42%. In contrast Cowling et al [23] observed that a school closure episode in Hong Kong in 2008 had little impact on influenza attack rates – although in that case school closure appears to have occurred after the epidemic peak. The largest scale study known to the authors that provides an estimate of the effectiveness of school closure on influenza epidemics is the work of Cauchemez et al [24]. Based on surveys of seasonal influenza during and between school terms in France, this work estimated that school closure could achieve at most a 17% reduction in attack rates, indicating that school closure may not be as effective as predicted our model.

## Conclusion

Our results suggest a critical role of combined social distancing measures in the potential control of a future pandemic. They indicate that non-pharmaceutical social distancing interventions are capable of preventing influenza epidemics with R_0 _values of up to 2.5, and of significantly reducing the rate of development and overall burden of epidemics with R_0 _values of up to 3.5, but only if used in combination, activated without delay, and maintained for a relatively long period. Our results also confirm the importance of rapid, decisive and robust action if social distancing interventions are to be useful in pandemic control. While such draconian measures seem unlikely to be mandated given their impact on personal freedom, they appear to have a key role to play in delaying the development of a "worst case" influenza epidemic (i.e. with a reproductive value of 3.5). They may be critical in holding back an epidemic until vaccines are deployed on a sufficient scale that subsequent relaxation of these rigorous measures will not result in a consequential acceleration in the scale of the outbreak.

## Competing interests

The authors declare that they have no completing interests.

## Authors' contributions

GM and JK were responsible for the conception and design of the simulation experiments. HK contributed epidemiological and public health expertise that informed model parameters. JK was responsible for software development and conducted simulation experiments. All authors were involved in analysis of simulation results and writing the manuscript.

## Pre-publication history

The pre-publication history for this paper can be accessed here:


